# Fluid Rheological Effects on the Flow of Polymer Solutions in a Contraction–Expansion Microchannel

**DOI:** 10.3390/mi11030278

**Published:** 2020-03-08

**Authors:** Purva P. Jagdale, Di Li, Xingchen Shao, Joshua B. Bostwick, Xiangchun Xuan

**Affiliations:** Department of Mechanical Engineering, Clemson University, Clemson, SC 29634-0921, USAdli2@clemson.edu (D.L.); xingchs@clemson.edu (X.S.); jbostwi@clemson.edu (J.B.B.)

**Keywords:** non-newtonian fluid, elasticity, shear thinning, inertia, microfluidics

## Abstract

A fundamental understanding of the flow of polymer solutions through the pore spaces of porous media is relevant and significant to enhanced oil recovery and groundwater remediation. We present in this work an experimental study of the fluid rheological effects on non-Newtonian flows in a simple laboratory model of the real-world pores—a rectangular sudden contraction–expansion microchannel. We test four different polymer solutions with varying rheological properties, including xanthan gum (XG), polyvinylpyrrolidone (PVP), polyethylene oxide (PEO), and polyacrylamide (PAA). We compare their flows against that of pure water at the Reynolds (Re) and Weissenburg (Wi) numbers that each span several orders of magnitude. We use particle streakline imaging to visualize the flow at the contraction–expansion region for a comprehensive investigation of both the sole and the combined effects of fluid shear thinning, elasticity and inertia. The observed flow regimes and vortex development in each of the tested fluids are summarized in the dimensionless Wi−Re and $\chi_{L}-Re$ parameter spaces, respectively, where $\chi_{L}$ is the normalized vortex length. We find that fluid inertia draws symmetric vortices downstream at the expansion part of the microchannel. Fluid shear thinning causes symmetric vortices upstream at the contraction part. The effect of fluid elasticity is, however, complicated to analyze because of perhaps the strong impact of polymer chemistry such as rigidity and length. Interestingly, we find that the downstream vortices in the flow of Newtonian water, shear-thinning XG and elastic PVP solutions collapse into one curve in the $\chi_{L}-Re$ space.

## 1. Introduction

Polymer solutions herein refer to aqueous solutions that contain dissolved high-molecular-weight polymers. They are non-Newtonian fluids exhibiting distinct rheological properties from Newtonian fluids such as elasticity and shear thinning or thickening [1,2]. Polymer solutions have been increasingly used in the past decade to manipulate particles and cells in microfluidic devices for various chemical and biomedical applications [3,4,5,6]. Moreover, they are frequently used in enhanced oil recovery and groundwater remediation to improve the displacement of trapped oil from a reservoir rock [7] or contaminants from a subsurface aquifer [8]. These applications benefit from a comprehensive understanding of the flow of polymer solutions through the pore spaces of porous media [9,10]. The real-world pores are highly heterogeneous and tortuous with successive contractions and expansions. They are often mimicked in laboratories by an abrupt rectangular contraction–expansion or a channel-centered obstacle as the simplest representation, which may be each repeated in a one-dimensional or two-dimensional array to model the flow through multiple inter-connecting pores [10,11]. The design and fabrication of these typically microscale pore structures can be readily implemented using photolithography-based microfluidic tools without much cost [12]. Moreover, the optical transparency of microfluidic devices fabricated in, for example, polydimethylsiloxane (PDMS) or poly(methyl methacrylate) (PMMA), enables pore-scale flow visualization using optical imaging [13,14,15]. 

The flow of polymer solutions in macroscopic channels with contractions has been extensively studied both experimentally and numerically [16]. These structures may be axisymmetric or planar with the characteristic length scale on the order of millimeters. In such geometries, the effects of fluid rheological properties (e.g., elasticity and/or shear thinning) are often coupled with and dominated by that of fluid inertia because of their macroscopic scale. Summaries of the published works on the entry flow of polymer solutions through macro-contractions can be found in [17,18]. The earliest attempts on such flows in micro-fabricated planar contraction–expansion geometries were made by Groisman and Quake [19,20] and later by Rodd et al. [21,22]. Since then, a number of other studies have been reported on the flow pattern of polymer solutions in contraction–expansion microchannels. The flow of a Newtonian fluid is highly three-dimensional with vortices formed downstream at the stagnant corner regions of the expansion part because of the fluid inertial effect [23,24]. In a Boger-like NaCl-added polyacrylamide (PAA) solution, elasticity-driven flow separation occurs upstream with the development of symmetric vortices at the corner regions of the contraction part [25]. Removing the salt makes a PAA solution highly shear thinning and elastic, where asymmetric and even unstable upstream vortices are observed when the fluid inertial effect still remains insignificant [26,27,28]. Similar flow patterns also appear in semi-dilute sodium hyaluronate [29] and surfactant [30] solutions that are both elastic and shear-thinning fluids. Moreover, they are observed in a blood-analog xanthan gum (XG) solution that has a much smaller relaxation time (i.e., much less elastic) than a PAA solution of similar shear rheology. However, fluid elasticity and inertia both take effects in the XG solution before the flow becomes unsteady [26]. In addition, upstream vortices (mostly asymmetric) develop in the flow of polyethylene oxide (PEO) solution [21,22,31,32], which has been utilized to enhance the microfluidic mixing [33,34,35]. 

In the previous studies on the flow of polymer solutions through contraction–expansion microchannels [19,20,21,22,23,24,25,26,27,28,29,30,31,32], the flow regimes and vortex growth have been each framed into a dimensionless parameter space, e.g., the Reynolds number (Re)-Weissenberg number (Wi) space. However, the majority of these studies tested the flow of one polymer solution only and compared it against that of water. Moreover, the tests were performed in contraction–expansion microchannels of varying geometries that differ in the contraction ratio (i.e., the width of the expansion part to that of the contraction part), aspect ratio (i.e., the width to depth ratio at the expansion part), or length of contraction etc. Therefore, the reported flow regimes and vortex growth in different polymer solutions may not be compared directly even though they are graphed into the same dimensionless parameter space. This is because of the strong dependence of the flow development of any fluids, no matter whether they are Newtonian or non-Newtonian, on the geometry of the contraction–expansion microchannel [10,11,19,20,21,22,23,24,25,26,27,28,29,30,31,32]. In this work we carry out an experimental study of the flow of four types of polymer solutions and compare it with that of water in the same contraction–expansion microchannel. These non-Newtonian fluids, which include the PAA, XG, PEO and polyvinylpyrrolidone (PVP) solutions, have different rheological properties. By varying the flow rate, we are able to attain Re and Wi that each span several orders of magnitude. Our aim is to obtain a fundamental knowledge of both the individual and combined effects of fluid elasticity, shear thinning and inertia on the flow of polymer solutions by comparing quantitatively their flow regimes and vortex growth in the same dimensionless parameter spaces. 

## 2. Experiment 

### 2.1. Microchannel 

Making a microchannel began with manufacturing of the mold which was undertaken by the soft-lithography method. In this method, a clean glass slide was first coated with photoresist (SU-8-25 Photoresist, MicroChem, Newton, MA, USA) with its thickness depending on the depth of microchannel one needs to achieve. This coating was done in steps by spinning the glass slide smeared with photoresist at various speeds for a certain amount of time in a spin coater (WS-400B-6NPP/LITE, Laurell Technologies, North Wales, PA, USA). After that, the glass slide was baked on hotplate (HP30A, Torrey Pines Scientific, San Marcos, CA, USA) at 65 °C for 5 min and 95 °C for 15 min. The next step in mold preparation was to mark the channel geometry on the glass slide, which was done with the help of a photo mask (CAD/Art Services Inc., Bandon, OR, USA). This masked glass slide was exposed to ultraviolet (UV) light of wavelength 350 nm (ABM Inc., San Jose, CA, USA) for a duration of 30 s. It was then baked at 65 °C for 1 min and 95 °C for 4 min. The microchannel mold was obtained by developing the glass slide in SU-8 developer solution (MicroChem, Newton, MA, USA) for 10 min and rinsed in isopropyl alcohol (Fisher Scientific, Fair Lawn, NJ, USA) followed by a hard bake at 150 °C for 5 min. It can be used multiple times.

**Figure 1:**
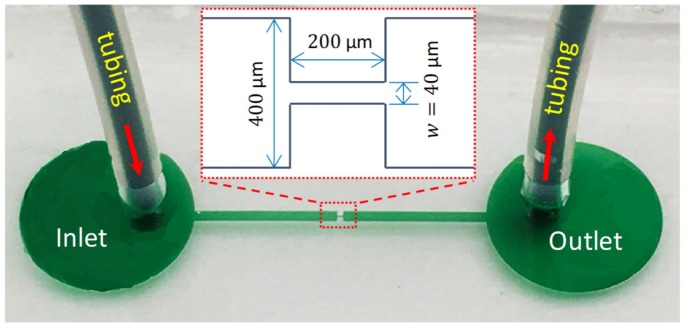
Picture of a fabricated 10:1:10 contraction- expansion microchannel with the inset displaying the dimensions.

A microchannel was made by pouring liquid PDMS, which was prepared by thoroughly mixing Sylgard 184 and its curing agent (Dow Corning, Midland, MI, USA) at a 10:1 ratio in weight, onto the mold placed in a petri dish. The liquid PDMS was degassed in an isotemp vacuum oven (13-262-280A, Fisher Scientific, Fair Lawn, NJ, USA) for 30 min, and then allowed to solidify in a gravity convection oven (13-246-506GA, Fisher Scientific, Fair Lawn, NJ, USA) for about 3 hours. After solidification, the chip was cut using a scalpel and peeled off from the petri dish. Through holes for the inlet and outlet were made in the PDMS with a metal punch. Any debris formed during the process were blown off with nitrogen gas. The channel side of the PDMS slab was irreversibly bonded to a clean glass slide immediately following a 1 min air-plasma treatment (PDC-32G, Harrick Scientific, Ossining, NY, USA). The chip was placed onto a hotplate for overnight baking to further enhance the bonding. A picture of the fabricated microchannel is shown in Figure 1. The channel is 1 cm long in total and 400 µm wide with a measured depth of 60 µm everywhere. It has a 200 µm long and 40 µm wide contraction in the middle, forming a 10:1:10 planar contraction–expansion part.

### 2.2. Fluids 

**Figure 2:**
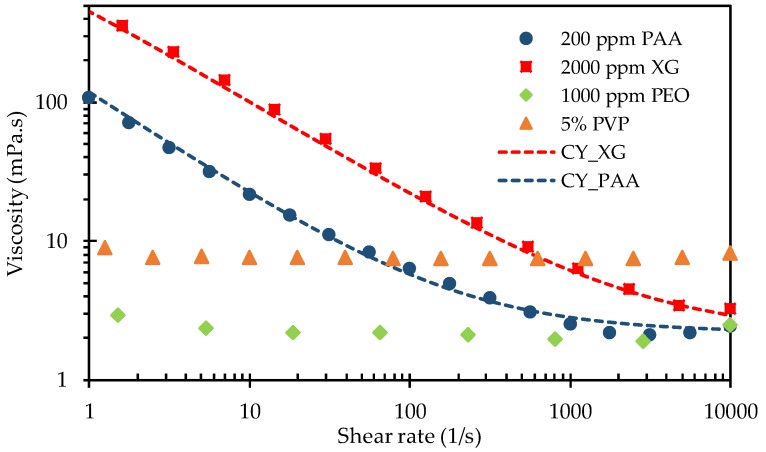
Experimentally measured viscosity data (symbols) of the prepared non-Newtonian fluids, where the dotted lines show the curve-fitting for the two shear-thinning xanthan gum (XG) and polyacrylamide (PAA) solutions using the Carreau–Yasuda model.

Five types of fluids were used, each having different rheological properties: Deionized (DI) water-Newtonian fluid; 5% wt. PVP solution (molecular weight, $M_{w}=0.36$ MDa, Sigma-Aldrich, St. Louis, MI, USA)—strongly elastic and negligibly shear-thinning fluid [36]; 2000 ppm XG solution (Tokyo Chemical Industry, Tokyo, Japan)—negligibly elastic and strongly shear-thinning fluid [37]; 1000 ppm PEO solution ($M_{w}=2$ MDa, Sigma-Aldrich, St. Louis, MI, USA)—mildly elastic and weakly shear-thinning fluid [21]; 200 ppm PAA solution ($M_{w}=18$ MDa, Polysciences, Warrington, PA, USA)—strongly elastic and strongly shear-thinning fluid [38]. The polymers, which were initially present in the granular powder form, were each combined with DI water to form a high-concentration solution. The desired concentrations of the polymers, as mentioned, were then obtained by diluting the high-concentration solutions with DI water. The dynamic viscosity of each of these prepared fluids was measured using a cone-plate rheometer (Anton Paar, MCR 302, Graz, Austria) over a wide range of shear rates at room temperature (varying from 23 to 24 °C). The viscosity data obtained are shown in Figure 2, where those of the shear-thinning XG and PAA solutions are each fitted using the Carreau-Yasuda model [39],

(1) $$\frac{\eta -\eta_{\infty }}{\eta_{0}-\eta_{\infty }}={\left[1+{\left(\lambda_{CY}\dot{\gamma }\right)}^{a}\right]}^{\left(n-1\right)/a}$$

In this equation, η is the fluid viscosity, $\eta_{\infty }$ is the infinite-shear-rate viscosity, $\eta_{0}$ is the zero-shear-rate viscosity, $\lambda_{CY}$ is a time constant, $\dot{\gamma }$ is the fluid shear rate, *a* is a fitting parameter, and n is the power-law index. Table 1 summarizes the rheological properties of the prepared fluids along with the curve-fitting values of the related parameters in Equation (1), where the relaxation times, λ, are extracted from the literature. 

The fluid inertial effect is characterized using the Reynolds number, Re, which was estimated at the contraction of the microchannel, i.e.,

(2) $$Re=\frac{\rho VD_{h}}{\eta \left(\bar{\dot{\gamma }}\right)}=\frac{2\rho Q}{\eta \left(\bar{\dot{\gamma }}\right)\left(w+h\right)}$$

where ρ is the fluid density assumed equal to the density of DI water, V is the average fluid velocity in the contraction, $D_{h}$ is the hydraulic diameter of the contraction, $\eta \left(\bar{\dot{\gamma }}\right)$ is the fluid viscosity estimated at the average fluid shear rate across the width, w, of the contraction, i.e., $\bar{\dot{\gamma }}=2V/w$, and h is the microchannel height. Typically the fluid inertia may be neglected unless Re≥1 [40]. The fluid elasticity effect is characterized by the Weissenberg number, Wi,

(3) $$Wi=\lambda \bar{\dot{\gamma }}=\frac{2\lambda Q}{w^{2}h}$$

which is a linear function of the fluid flow rate. The relative contribution between the fluid inertial and elastic effects is measured by the elasticity number, El,

(4) $$El=\frac{Wi}{Re}=\frac{\lambda \eta \left(\dot{\gamma }\right)\left(w+h\right)}{\rho w^{2}h}$$

which is still a function of kinematics for shear thinning fluids. To facilitate the description, we define in this study a fluid as strongly elastic if El≥10, mildly elastic if 10>El≥1, and weakly elastic if 1>El>0. The fluid shear-thinning effect is characterized by the power-law index, n, shown in Table 1, where a smaller value indicates a stronger shear thinning effect. Following Lindner et al. [41], we define here a fluid as strongly shear thinning if n<0.65. 

### 2.3. Method

The contraction–expansion region of the microchannel, as highlighted by the rectangle in Figure 1, is the region of interest as the effects of contraction are most noticeable here. To visualize the flow pattern in this region, polystyrene particles (Bangs Laboratories) of diameter d=0.995 µm were added to each solution. Their small size makes sure that they follow the fluid streamlines because of the negligible particle inertia. This is evidenced by the small particle Reynolds number,

(5) $$Re_{p}=Re{\left(\frac{d}{D_{h}}\right)}^{2}$$

which remains smaller than 0.1 even at the highest flow rate of 50 mL/h in our tests. The particle-fluid mixture was driven through the contraction–expansion microchannel with the help of a syringe pump via an air-tight glass syringe (KD Scientific, Holliston, MA, USA). Plastic tubes were inserted into the holes that were punched in the microchannel during the fabrication to carry the test fluid into and out of the channel. As the channel geometry before and after the contraction is exactly the same, the orientation of the channel does not affect the experimental results. The behavior of tracing particles at the contraction–expansion region was recorded using an inverted fluorescent microscope (Nikon Eclipse TE2000U, Nikon Instrument, Lewisville, TX, USA) with a charge-coupled device (CCD) camera (Nikon DS-Qi1Mc, Nikon Instrument, Lewisville, TX, USA) at a rate of around 15 frames per second. The obtained digital images were processed using the Nikon imaging software (NIS-Elements, Nikon Instrument, Lewisville, TX, USA). 

## 3. Results and Discussion

### 3.1. Effects of Inertia 

**Figure 3:**
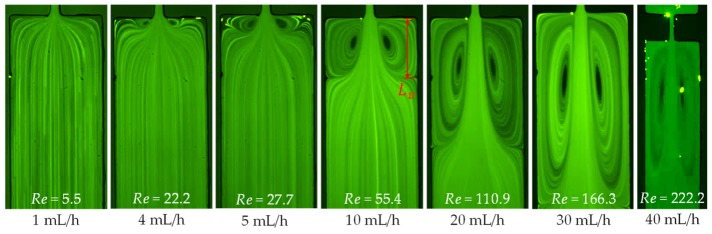
Inertial flow (downward) behavior of Newtonian water at the expansion part of the microchannel in a range of flow rates. No circulations are formed at the contraction part in any of the tested flow rates. The scale bars each represent 100 µm.

The sole effect of inertia on flow in the contraction–expansion microchannel was studied using DI water. Figure 3 shows the flow patterns at different values of Reynolds number. For a relatively small Reynolds number (Re<10), the flow is symmetric about the contraction along the length of the microchannel. As the flow rate increases to around 3 mL/h with Re~15, a pair of identical lip vortices start forming downstream at the two re-entrant corners, right where the expansion part begins. Their sizes grow along both the width and length directions of the microchannel as the Reynolds number increases, showing the effect of increasing inertia. Once the lip vortices hit the salient corners of the expansion part at around 6 mL/h (Re=33.3), what are then called corner vortices continue growing in length downstream as the Reynolds number increases further (Re>55). These vortices still remain symmetric at a very high Reynolds number of up to 222.2 in our experiment (at 40 mL/h) as seen in Figure 3. It is, however, noted that the symmetry of the corner vortices is highly dependent on the aspect ratio (width/depth) of the microchannel. As demonstrated by Oliveira et.al [22], the inertial flow through a contraction–expansion microchannel may become asymmetric about the channel axis when the aspect ratio is large and/or the Reynolds number is very high. Our current experimental results are in good agreement with the analysis presented in the paper as far as the range of Reynolds number and aspect ratio are concerned. The channel lengthwise dimension of the corner vortices is quantified by $L_{v}$ as highlighted on the image in Figure 3, which will be presented in Section 3.6 below.

### 3.2. Effects of Elasticity and Inertia 

**Figure 4:**
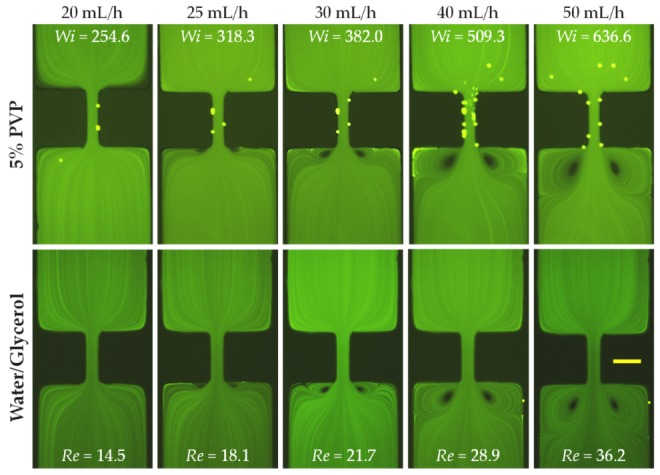
Comparison of the inertial flows (downward) of the viscosity-matched elastic polyvinylpyrrolidone (PVP, 5% in weight, top row) and Newtonian water/glycerol (40/60 in weight, bottom row) solutions at the contraction–expansion region of the microchannel. The scale bar represents 100 µm.

The prepared PVP (5% in weight) solution is a highly elastic (with an elasticity number, El=17.2), and highly viscous fluid. It may be safely viewed as a Boger fluid [42] as its measured viscosity does not change over a large range of shear rates (Figure 2). Therefore, this solution allows us to study both the sole effect of fluid elasticity and the combined effect of fluid elasticity and inertia. The flow of viscoelastic PVP solution in the contraction–expansion microchannel is shown in Figure 4 (top row). No circulations are observed until the flow rate reaches 20 mL/h, at which the Reynolds number becomes Re~15. This value seems consistent with the threshold flow rate for the onset of vortices in DI water (Figure 3). Moreover, the vortex growth pattern is similar to the case of DI water (Figure 3), starting from the formation of lip vortices to the growth of lip vortices, the formation of corner vortices and then the growth of corner vortices at the extraction part of the microchannel. Interestingly, while the formation of fluid circulations is significantly delayed in the PVP solution (about 20 mL/h) as compared to the pure water case (about 3 mL/h), the sizes of the vortices in the two fluids are visually very similar if compared at the same Reynolds number (e.g., 4 mL/h in water with Re=22.2 in Figure 3 vs. 30 mL/h in PVP with Re=21.7 in Figure 4). These observations make us hypothesize that the strong elasticity of the PVP solution (with an elasticity number, El=17.2, Table 1) may have little influences on the flow pattern. Or at least the influence of the elasticity effect is much smaller than that of the fluid inertial effect even though the Weissenberg number, Wi, can reach the order of 1000. To further verify this hypothesis, we studied the flow of a Newtonian water/glycerol (40/60 in weight) solution, whose kinematic viscosity [43] nearly matches that of the PVP solution, through the contraction–expansion microchannel (Figure 4, bottom row). Indeed, in the entire range of tested flow rates, the flow patterns are visually identical in between the viscoelastic and Newtonian fluids at the same flow rates and in turn the same values of Reynolds number. We speculate that the reason behind such an insignificant fluid elasticity effect on the flow pattern may be associated with the relatively short PVP polymers, such that their extensional stretching and re-orientation do not have a strong impact on the fluid motion at the contraction–expansion region.

### 3.3. Effects of Shear Thinning and Inertia

**Figure 5:**
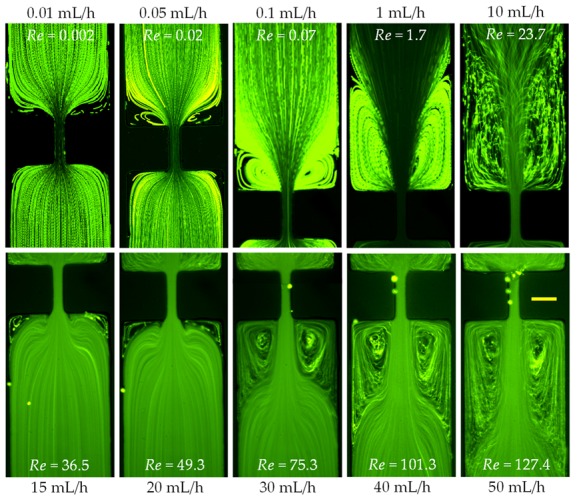
Inertialess (Re<0.1) or inertial flow (downward) of 2000 ppm shear-thinning XG solution at the contraction–expansion region of the microchannel. The scale bar represents 100 µm.

The prepared XG solution (2000 ppm) is a purely shear-thinning fluid (power-law index, n=0.32) with negligible elasticity [41,44]. This solution thus allows us to study the effect of fluid shear thinning in both an inertialess (say, Re<0.1) and an inertia flow. Figure 5 shows the flow patterns at the contraction–expansion region of the microchannel in a range of flow rates spanning three orders of magnitude. At a flow rate as low as 0.01 mL/h (where Re=0.002), a pair of small lip vortices already start appearing upstream at the re-entrant corners of the contraction part while the flow downstream in the expansion part is still Newtonian-like. This observation is opposite the purely elastic (PVP in Figure 4) or the Newtonian (water in Figure 3) case discussed earlier, where the lip vortices first appear downstream at the internal corners of the expansion part when the Reynolds number reaches a value of Re~15. With the increase of Reynolds number, the lip vortices in the shear-thinning XG solution quickly extend both outward to the sidewalls of the channel (i.e., forming the corner vortices) and upward opposing the flow. These continuously expanded upstream vortices remain approximately symmetric in the whole range of tested flow rates (up to 50 mL/h). They should be a direct consequence of the strong shear thinning effect of the XG solution. When the Reynolds number increases to about Re=23.7 (10 mL/h), the streamlines start to bend in the expansion part of the microchannel because of the fluid inertial effect. Further increasing the Reynolds number to Re=36.5 (15 mL/h) causes the formation of two small vortices at the stagnant salient corners of the expansion part. Similar to those in the flow of water (Figure 3) and PVP (Figure 4) solutions, these symmetric downstream vortices can be seen to grow with the increase of Reynolds number both inward from the corners to the center of the channel and downward following the flow. Moreover, the length of such inertially formed corner vortices in the XG solution seems to be comparable to that in water if at the same Reynolds number, especially when the Reynolds number is high. For example, the lengthwise dimension of the downstream vortex is $L_{v}=544$ µm at Re=110.9 (20 mL/h) in water as compared to $L_{v}=516$ µm at Re=101.3 (40 mL/h) in the XG solution. A complete comparison of the vortex lengths in the two fluids will be presented in Section 3.6. 

### 3.4. Effects of Elasticity, Shear Thinning and Inertia

**Figure 6:**
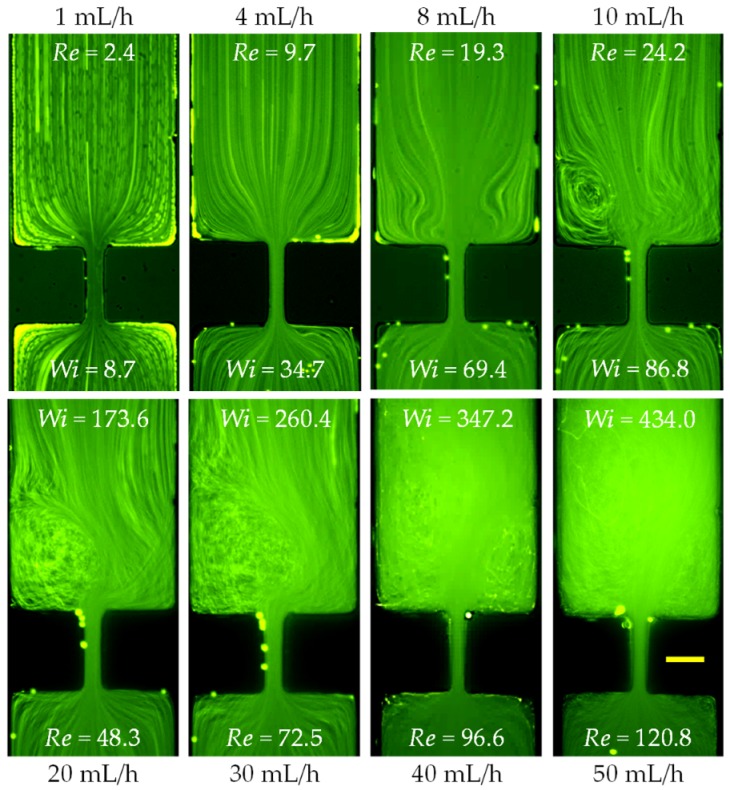
Inertial flow (downward) of 1000 ppm mildly elastic and weakly shear-thinning polyethylene oxide (PEO) solution at the contraction–expansion region of the microchannel. The scale bar represents 100 µm.

The prepared PEO solution (1000 ppm) is a mildly elastic fluid (with an elasticity number, El=3.6) fluid. Similar to the strongly elastic 5% PVP solution (El=17.2), this solution also has a weak shear thinning effect (the fitted power-law index to the experimental data is n=0.97). However, it has a much smaller viscosity than the PVP solution (Table 1), yielding a much larger value of Reynolds number at the same flow rate. Figure 6 shows the flow of PEO solution through the contraction–expansion region of the microchannel. At a flow rate smaller than about 3 mL/h, the PEO solution behaves like pure water (Figure 3) with a symmetric flow pattern in both the length and width directions of the contraction–expansion region. Increasing the flow rate to around 4 mL/h (where the Reynolds number is Re=9.7 and the Weissenberg number is Wi=34.7), the streamlines upstream in the contraction part start to bend. At a higher Reynolds number, the bending and diverging of the streamlines in the upstream region becomes more evident but remains symmetric until Re=24.2 is reached at the flow rate of 10 mL/h, where the bent streamlines collapse into a single vortex on one side of the wall near the stagnant salient corner of the contraction part. On further increasing the Reynolds number, the size of the single vortex increases along the length of the channel and the bending of streamlines continues on the other side. At Re=96.6 (40 mL/h), another small vortex is formed upstream in the opposite corner of the channel at the contraction part while the initial large vortex continues growing. In this whole range of flow rates, no inertial circulations are observed downstream at the expansion part of the microchannel like those in the Newtonian water (Figure 3), viscoelastic PVP (Figure 4) and shear-thinning XG (Figure 5) cases, although the streamlines appear to be bent to some extent. As the PVP solution, which has a much stronger elasticity than the PEO solution, exhibits similar behaviors to water if at the same Reynolds number, we attribute the observed anomalous flow pattern in the PEO solution to the significantly greater extensional stretching and re-orientation of long PEO polymers ($M_{w}=2$ MDa) at the contraction–expansion region. This is because the average length of a polymer chain is directly related to its molecular weight. 

### 3.5. Effects of Elasticity, Shear Thinning, Inertia and More

**Figure 7:**
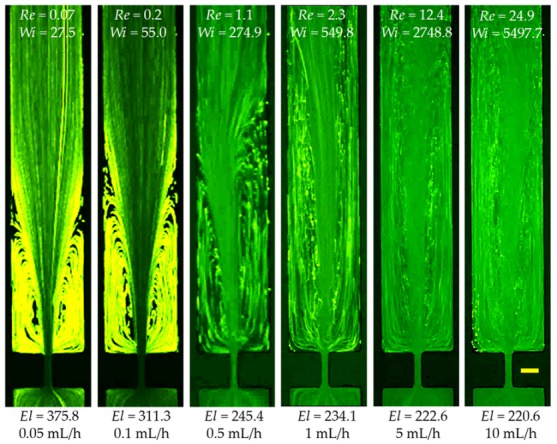
Inertialess (Re<0.1) or inertial flow (downward) of 200 ppm highly elastic and highly shear thinning PAA solution at the contraction–expansion region of the microchannel. The scale bar represents 100 µm.

**Figure 8:**
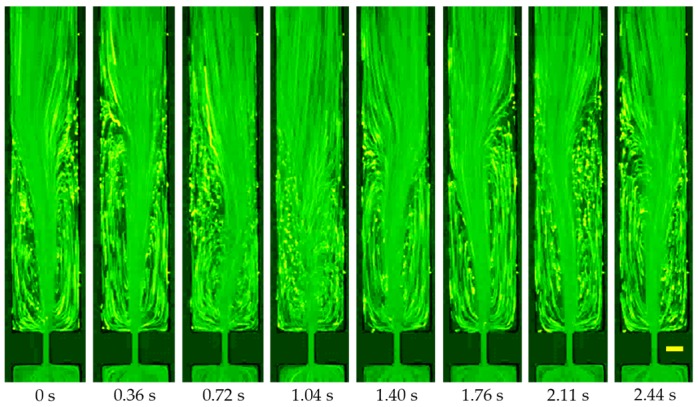
Demonstration of the unstable vortices in the flow of 200 ppm PAA solution at the contraction part of the microchannel under a flow rate of 0.5 mL/h. The scale bar represents 100 µm.

The prepared PAA solution (200 ppm) is a highly elastic (El~200) and highly shear-thinning (power law index, n=0.37) fluid. This solution also possesses a non-negligible second normal stress difference that has been demonstrated to cause secondary flow over the channel cross-section. Moreover, the length of PAA polymers ($M_{w}=18$ MDa) should be much larger than that of PEO polymers, which is supposed to have an even greater influence on the flow pattern than the latter. Figure 7 shows the flow of PAA solution in the contraction–expansion microchannel. In the absence of fluid inertial effect (Re<0.1), the flow pattern is similar to that observed in the XG solution (Figure 5) due to a dominant fluid shear-thinning effect. As the Reynolds number increases, lip vortices first occur upstream at the re-entrant corners and then grow into corner vortices at the salient corners of the contraction part. These symmetric vortices, however, extend much further upward than those in the XG solution. Moreover, the streamlines bend and the flow becomes unstable at the contraction part when the Reynolds number increases to Re~1 (see 0.5 mL/h in Figure 7). As illustrated by the sequential images in Figure 8, the upstream vortices oscillate from large to small lengths and become strongly asymmetric. They can collapse and reappear, continuing to oscillate further without an apparent period. These observations are distinctly different from the flow of XG solution in Figure 5, which might be associated with both the long PAA polymers and the second normal stress difference. The upstream vortices seen in Figure 7 grow very large in size as the Reynolds number is increased such that they could not fit into our observation window. 

**Figure 9:**
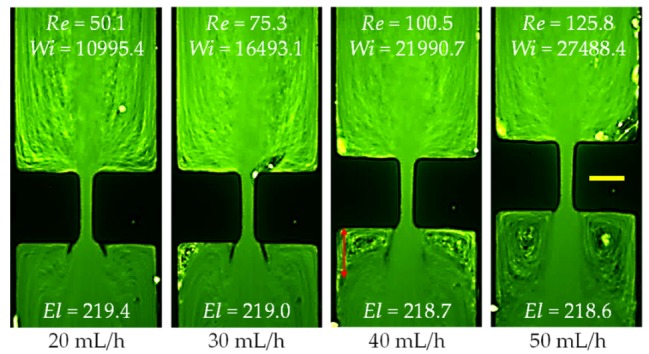
Strongly inertial flow (downward) of 200 ppm highly elastic and highly shear thinning PAA solution at the contraction–expansion region of the microchannel. The scale bar represents 100 µm.

Similar to the XG solution, the streamlines in the PAA solution also bend at the expansion part of the microchannel. Moreover, corner vortices occur downstream at Re~75 (30 mL/h) and grow with the further increase of the Reynolds number as viewed from Figure 9. However, these downstream vortices take place at a larger Reynolds number than in the XG solution. Moreover, they seem to be slightly asymmetric with the size being significantly smaller than in the XG solution or pure water at a similar Reynolds number. It is again speculated that the long PAA polymers and second normal stress difference may be both responsible for the observed flow phenomena in Figure 9. We are, however, unsure if the extremely large elasticity of the PAA solution, whose Weissenberg and elasticity numbers are each about 1.5–2 orders of magnitude greater than the PEO solution, may play a role in the observed flow instabilities in Figure 7, Figure 8 and Figure 9.

### 3.6. Summary of Flow Regimes and Vortex Development

**Figure 10:**
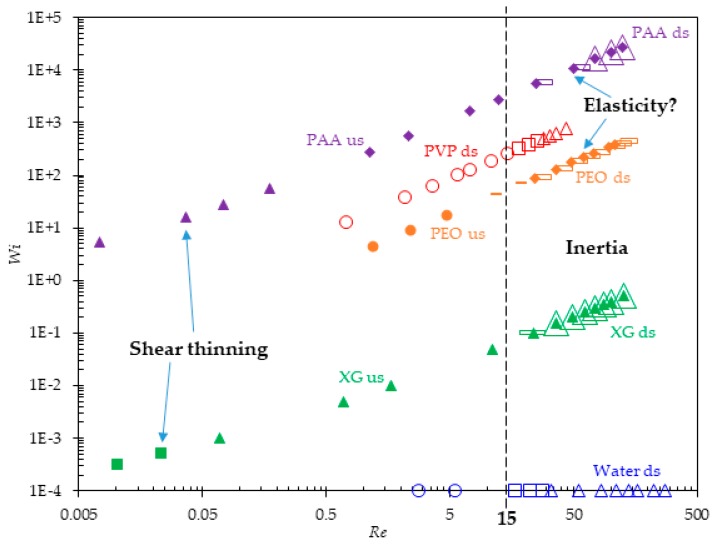
Summary of flow regimes in the Wi−Re space for the flow of Newtonian and non-Newtonian fluids through a contraction–expansion microchannel. Filled markers are for the upstream (us) flows at the contraction part and hollow markers are for the downstream (ds) flows at the expansion part of the microchannel: circles for no bending streamlines or vortices; squares for stable lip vortices; triangles for stable corner vortices; dashes for bending streamlines; diamonds for unstable or asymmetric corner vortices. The vertical dashed line indicates the Reynolds number, Re=15, at which the fluid inertia starts drawing apparent disturbances to the flow at the expansion of the microchannel. Note that the Weissenberg number has been assumed to be 0.01 and 0.1 for the pure water and XG solution, respectively, for the purpose of the graph.

**Figure 11:**
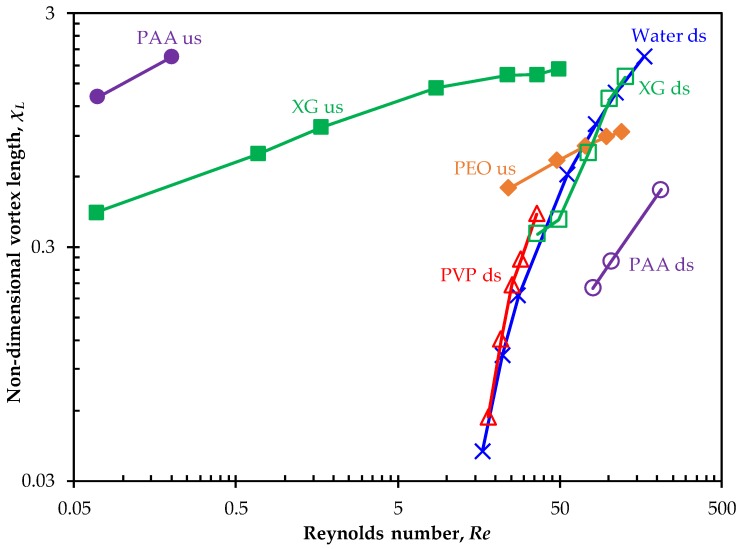
Non-dimensional vortex length, $\chi_{L}=L_{v}/w$, against the Reynolds number in the flow of Newtonian and non-Newtonian fluids through a contraction–expansion microchannel. Filled markers are for the upstream (us) vortices at the contraction part and hollow markers are for the downstream (ds) vortices at the expansion part of the microchannel. Note that only the size of stable vortex (either symmetric or asymmetric) is shown in the plot.

Figure 10 summarizes the observed flow regimes of the prepared solutions in the Wi−Re space. Note that in order to use the logarithmic scale for the vertical axis, we set the Weissenberg number of Newtonian water to a very small value, $Wi=10^{-4}$, regardless of flow rate. We also set the Weissenberg number of the negligibly elastic XG solution at the flow rate of 0.01 mL/h to the same value, based on which Wi at higher flow rates can be calculated using Equation (3). As highlighted by the vertical dashed line in Figure 10, disturbances to the downstream flow at the expansion part of the microchannel (see the hollow markers) initiate at about Re=15 in all fluids because of the inertial effect. They start with small lip vortices in water and the PVP solution while first appearing as bending streamlines in the other solutions. They all develop into stable corner vortices except in the PEO solution where the bent of streamlines grows slowly with the increase of Reynolds number to Re~120. However, the threshold Reynolds number at which the downstream corner vortices first occur in the PAA solution (Re~75) is approximately twice of that in water and the PVP and XG solutions (Re~35). Such a suppression of inertial vortices may be associated with the stretching and re-orientation of long flexible PEO or PAA polymers at the channel contraction region, as compared to the short PVP or rigid XG polymers. Disturbances to the upstream flow at the contraction part of the microchannel (see the filled markers in Figure 11) take place very early (Re<0.01) in the strongly shear-thinning XG and PAA solutions. They both start from small lip vortices that quickly expand to form stable corner vortices when the fluid inertia is still negligible (Re<0.1). Such a development of the fluid shear-thinning induced upstream vortices is similar to that of the inertially formed downstream vortices in water and the PVP solution. In contrast, upstream disturbances do not occur in the PEO solution until the Reynolds number reaches the value of Re~10. They further develop into asymmetric corner vortices at Re≥24, which is speculated to share a similar origin to the unstable corner vortices formed in the PAA solution at Re≥1 because of the reasons stated above.

Vortex development in the prepared solutions can be quantified by measuring the length of stable vortex, $L_{v}$ (highlighted in Figure 3), at either the upstream contraction or the downstream expansion of the microchannel as it grows with the increase of flow rate. Figure 11 plots the normalized (by the width of the channel, $w_{ch}$) vortex length, $\chi_{L}=L_{v}/w_{ch}$, in each of the prepared solutions against the Reynolds number. The downstream vortices in the flow of water (16.7≤Re≤222.2) and PVP (18.1≤Re≤36.2) solutions collapse into one curve in the dimensionless $\chi_{L}-Re$ plot, which also coincides with that for the downstream vortices in the inertial flow of XG solution (36.5≤Re≤127.4). The downstream vortices in the PAA solution are much smaller than those formed in the Newtonian water, elastic PVP and shear-thinning XG cases at similar values of Reynolds number. On the contrary, the upstream vortices in the PAA solution have the largest sizes especially when the fluid inertial effect is not significant (Re<1). Those in the XG solution are greater than the upstream asymmetric vortex (or vortices) in the PEO solution that occur at 24.2≤Re≤120.8. 

### 3.7. Comparison with Other Polymer Solutions 

The flows of several other types of polymer solutions in planar contraction and/or expansion microchannels have been experimentally studied in the literature. These solutions can be divided into two groups: one group has similar rheological properties to the viscoelastic PVP and PEO solutions that has a negligible (i.e., Boger fluid) or weak (i.e., Boger-like fluid) shear thinning effect, and the other group has similar rheological properties to the PAA solution that has strong elasticity and strong shear-thinning effects. For the latter group, symmetric vortices are reported in the flow of hyaluronic acid sodium salt (Na-HA) [29], cetyltrimethylammonium bromide (CTAB) and cetylpyridinium chloride (CPyCl) surfactant [30], DNA [45,46], and poly (octadecyl methacrylate) (PODMA) [47] solutions through an abrupt planar contraction when the fluid inertia is weak. Increasing the flow rate makes the vortices asymmetric and even unstable, which is consistent with our observations in the PAA solution (Figure 7 and Figure 8). Moreover, the size of the fluid vortices in the expansion flow of PODMA solution are found to be significantly smaller than those in the Newtonian arachidyl alcohol at the same flow rate [47]. This phenomenon is also apparent in our test with the PAA solution (Figure 8 and Figure 11). For the group of Boger or Boger-like fluids, a Newtonian-like contraction flow is reported in both the low-concentration atactic polystyrene (aPS) solution [48] and the PEO solution mixed with a low-concentration sodium dodecyl sulfate (SDS) surfactant [49]. Such a flow, which is consistent with our observations in both the PVP (Figure 4) and PEO (Figure 6) solutions at small flow rates, further develops with elastic instabilities at higher flow rates like those observed in the PEO solution (Figure 6). Increasing the concentration of aPS or SDS enhances both the fluid shear thinning and elasticity effects, leading to firstly fluid vortices and then unstable flows with the increase of flow rate [48,49]. This trend resembles our observations in the PAA solution (Figure 7, Figure 8 and Figure 9). In another interesting study, symmetric vortices are induced upstream in the flow of NaCl-added Boger-like PAA solutions through a hyperbolic contraction when Re reaches the order of unity [25]. Their sizes grow with the increase of flow rate and/or PAA concentration (which enhances the fluid shear thinning and elasticity effects). This study suggests that the combination of fluid inertia and elasticity may also draw vortices in a contraction flow like those in the strongly shear-thinning XG (Figure 3) and PAA (Figure 7) solutions.

## 4. Conclusions

We have experimentally investigated the fluid rheological effects on the flow of four different water-based polymer solutions, i.e., XG, PVP, PEO and PAA, through a rectangular contraction–expansion microchannel. We have compared the flow regimes and vortex growth developments both among these polymer solutions and with pure water in a very wide range of Reynolds numbers and Weissenburg numbers. We have also compared our experimental observations with those of other types of polymer solutions available in the literature. In summary, we find that the fluid inertia alone induces symmetric vortices downstream (in the Newtonian water) at the expansion part of the microchannel. The fluid shear thinning alone draws symmetric vortices upstream (in the inelastic XG solution) at the contraction part of the microchannel. The fluid elasticity alone does not cause any visible disturbances to the inertialess flow of Boger-like (PVP, PEO or Nacl-added PAA [25]) solutions. The combination of fluid inertia with shear thinning expands the upstream vortices (in the XG solution) and also induces symmetric Newtonian-like vortices downstream. The combination of fluid inertia with elasticity yields significantly different polymer-dependent flow patterns in the Boger-like (PVP, PEO and Nacl-added PAA [25]) solutions. The combination of fluid elasticity with shear thinning leads to greatly enhanced and even unstable upstream vortices (in the strongly viscoelastic and strongly shear-thinning PAA solution) as compared to the symmetric vortices caused by the shear-thinning effect alone (in the XG solution). The combination of fluid inertia with elasticity and shear thinning leads to significantly extended unstable upstream vortices and strongly suppressed downstream vortices (in the PAA solution). Our work indicates that the effect of fluid elasticity requires further intensive studies, which may be performed with PEO solutions of varying molecular weights and/or PAA solutions of varying shear thinning effects in contraction–expansion microchannels with varying contraction and aspect ratios.

## Figures and Tables

**Table 1:** Rheological properties of the prepared fluids. Note the elasticity number, El, was calculated at the flow rate of 10 mL/h.

Fluid	$\eta_{0}(\mathrm{mPa}\cdot s)$	$\eta_{\infty }\;(\mathrm{mPa}\cdot s)$	$\lambda_{CY}(s)$	a	*n*	λ	El
DI Water	1.0	1.0	1	−	1	0	0
5% PVP	7.6	7.6	1	−	~1	2.2 *	17.2
2000 ppm XG	1870	2.1	6.62	1.02	0.32	~0	~0
1000 ppm PEO	2.3	2.3	−	−	~1	1.5 **	3.6
200 ppm PAA	4900	2.2	111.1	1.2	0.37	95 ***	220.6

* Liu et al. [37]; ** Rodd et al. [21]; *** Poole and Escudier [38].
